# A retrospective study of microwave ablation and thoracoscopic surgery for multiple primary lung cancer: a propensity score matching analysis

**DOI:** 10.3389/fsurg.2025.1547048

**Published:** 2025-03-11

**Authors:** Bangsheng Li, Shengguai Gao, Jie Mao, Zhenghong Yang, Ying Chen, Xi Wang, Yunchao Huang

**Affiliations:** ^1^ Department of Thoracic Surgery, Yunnan Cancer Hospital, The Third Affiliated Hospital of Kunming Medical University, Peking University Cancer Hospital Yunnan, Kunming, China; ^2^ Department of Thoracic Tumor Center, Northeast Yunnan Central Hospital, Zhaotong, Yunnan, China

**Keywords:** multiple primary lung cancer, video-assisted thoracoscopic surgery, microwave ablation, complication, pulmonary function

## Abstract

**Purpose:**

Microwave ablation (MWA) is a minimally invasive local treatment with demonstrated safety and efficacy, but its role in managing multiple primary lung cancer (MPLC) is not well-established. This study retrospectively evaluates the clinical effectiveness of MWA compared to video-assisted thoracoscopic surgery (VATS) in treating MPLC.

**Materials and methods:**

A retrospective analysis was conducted using data from patients with non-small cell lung cancer (NSCLC) treated at Peking University Cancer Hospital Yunnan Hospital between January 2021 and April 2024. All patients had undergone surgical resection for their first primary lung cancer (FPLC) and subsequently received either MWA or VATS for second primary lung cancer (SPLC). After 1:1 propensity score matching (PSM), 202 patients per group were included. Study endpoints included progression-free survival (PFS), overall survival (OS), complications, and pulmonary function changes.

**Results:**

Median follow-up was 24.47 months. Survival analysis revealed a statistically significant difference in PFS between MWA and VATS groups (HR = 2.74, 95% CI: 1.40–5.36, *p* = 0.006), while OS showed no difference (HR = 1.41, 95% CI: 0.45–4.36, *p* = 0.56). The incidence of grade ≥ II complications was significantly lower in the MWA group (*p* < 0.001). Pulmonary function tests indicated no significant changes in forced vital capacity (FVC), forced expiratory volume in 1 s (FEV1), FEV1%, maximal voluntary ventilation (MVV), and diffusion capacity of the lung for carbon monoxide%(DLCO%) before and 1–3 month post MWA (*p* > 0.05).

**Conclusions:**

In MPLC patients with stage IA SPLC, VATS demonstrates a greater clinical efficacy advantage in terms of local tumor control compared to MWA. Additionally, MWA provided significant advantages in reducing complication severity and preserving pulmonary function. These findings suggest that the therapeutic approach combining surgery with MWA represents a safe and effective option for MPLC.

## 1 Introduction

With the advent of the post-pandemic era, the application of chest CT in lung cancer screening has become increasingly widespread. Meanwhile, the detection rate of MPLC has risen significantly across various countries and regions, accounting for approximately 3%–13% of all lung cancer cases (1, 2). In 1924, Beyreuther first reported MPLC, which has since garnered clinical attention (3). MPLC refers to the simultaneous or sequential discovery of two or more primary cancer lesions in the lungs that are anatomically distinct and originate independently (4). In clinical practice, most MPLC cases are diagnosed as early-stage lung cancer (5). Therefore, pulmonary resection remains the gold standard treatment (6). However, for MPLC patients who have already undergone one surgical procedure, even sublobar resection inevitably sacrifices substantial lung tissue, potentially increasing perioperative morbidity and mortality and affecting long-term quality of life (7, 8). Therefore, stereotactic body radiotherapy (SBRT) and ablation therapy play a pivotal role.

SBRT has become the standard treatment for early-stage patients who are ineligible for surgery, offering comparable overall survival and local control rates to surgical interventions (9). Nevertheless, severe adverse effects such as pneumonitis and bone marrow suppression remain significant concerns, and its application in multiple lesions is limited (10). On the contrary, local ablation therapy offers substantial advantages in avoiding radiation-associated toxicity and managing various lesions (11). MWA uses microwave energy to rapidly heat target tissues to 60°C–150°C, causing irreversible damage and necrosis to cancer cells. Compared to radiofrequency ablation (RFA), MWA has a shorter ablation time, larger ablation zones, and reduced heat sink effects (12). Existing data suggest that MWA demonstrates unique advantages in the treatment of early-stage NSCLC (13–15).

This study aims to retrospectively compare the short-term efficacy, complication rates, and changes in pulmonary function between MWA and VATS in treating SPLC presenting as stage IA.

## 2 Materials and methods

### 2.1 Patients and data collection

From January 2021 to April 2024, patients with lung cancer who underwent surgical resection for FPLC and received MWA or VATS for SPLC in Peking University Cancer Hospital Yunnan Hospital were retrospectively investigated. The study received approval from the hospital's ethics committee.

**Figure 1:**
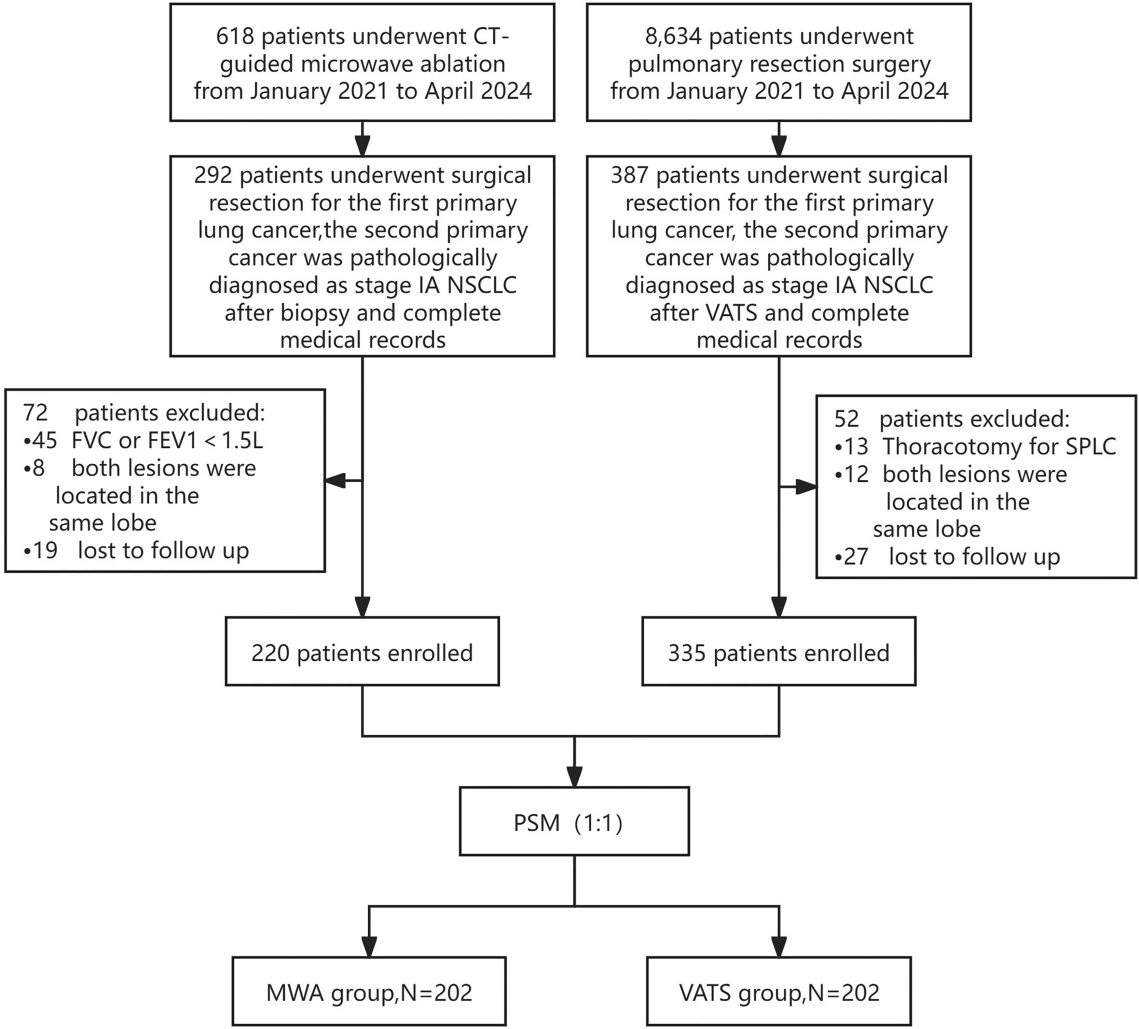
Flow chart of patient enrollment.

Before treatment, medical history collection, physical examination, and imaging studies were performed. The optimal treatment plan was determined after a multidisciplinary consultation involving thoracic surgeons, medical oncologists, radiologists, respiratory physicians, and pathologists. Inclusion criteria: (1) Patients with FPLC who underwent surgical resection and were diagnosed with stage I-IIIB NSCLC; (2) SPLC lesions with a diameter ≤3 cm as shown by CT or PET-CT; (3) Lesions located in bilateral lungs or different lobes of the same lung; (4) SPLC confirmed as NSCLC via surgery, bronchoscopy, or concurrent biopsy during ablation. Exclusion criteria: (1) Evidence of mediastinal lymph node or distant metastasis detected by CT, PET-CT, MRI, or ultrasound; (2) SPLC treated with open thoracotomy; (3) FVC or FEV1 less than 1.5l before the second treatment; (4) Incomplete medical records. Patients were matched at a 1:1 ratio using propensity score matching (PSM) based on sex, age, TNM stage of FPLC, FVC before the second treatment, FEV1 before the second treatment, and SPLC lesion size. The patient selection flowchart is shown in Figure 1. Detailed clinical data were collected from medical records, including sex, age, body mass index, smoking history, past medical history, TNM stage, lesion size and consolidation tumor ratio (CTR), histological type, complications, and pulmonary function indices.

**Figure 2:**
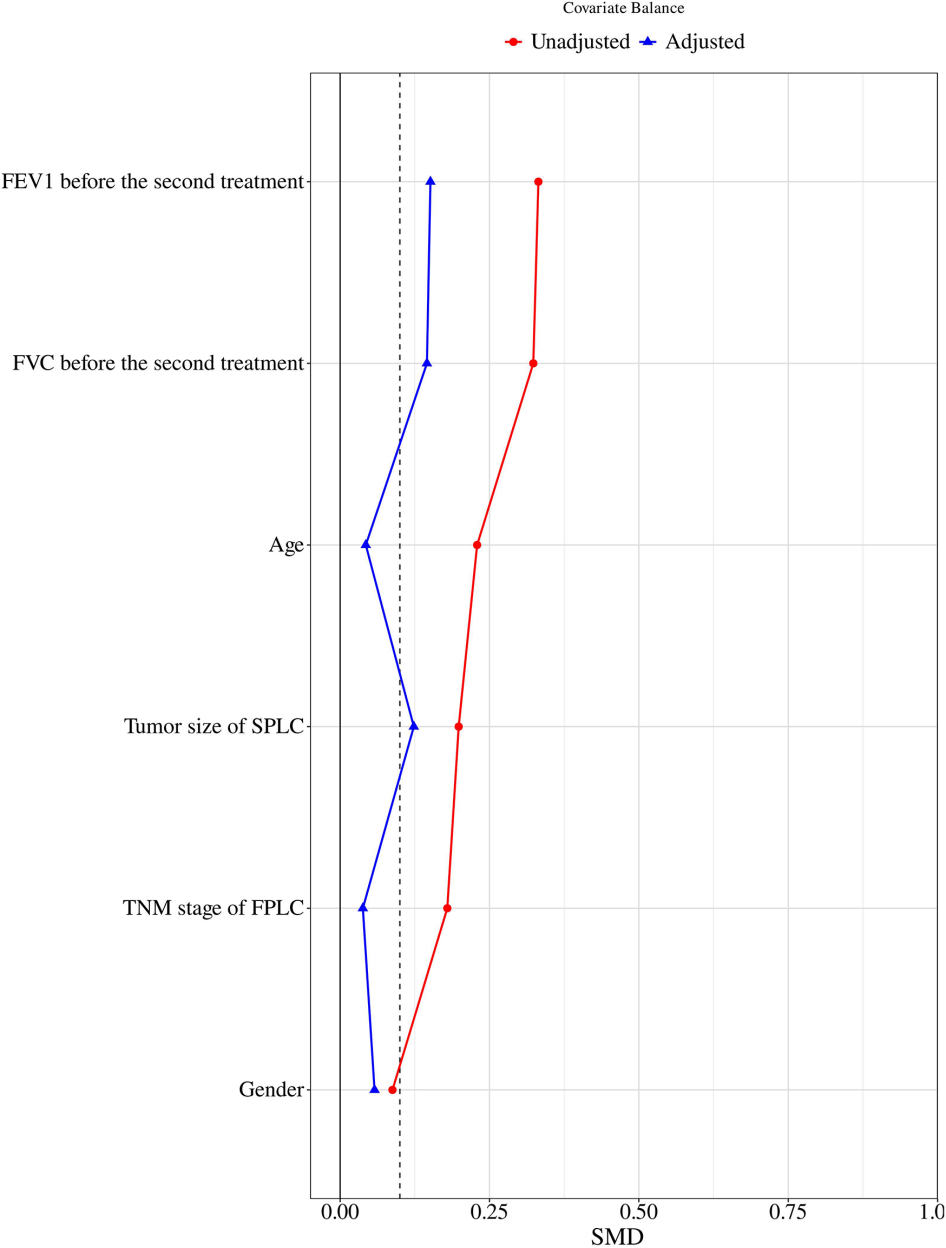
Standardized mean difference of variables before and after PSM.

### 2.2 Instrument and MWA procedure

This study utilized the KY-2000 microwave ablation system with a working frequency of 2,450 ± 50 MHz and an output power range of 0–100 W for ablation treatment. The ablation needles had an effective length of 100–180 mm and external diameters of 16G, 18G, and 19G. A water circulation cooling system maintained the surface temperature of the needles. The ablation process was guided by a Siemens Definition AS + spiral CT.

A personalized MWA treatment plan was developed preoperatively based on the patient's medical history and examination results. Patients were fully informed about the treatment process and associated risks, and written informed consent was obtained. The appropriate body position was determined according to the lesion's location, and the optimal puncture site was identified using a metal surface locator. All MWA procedures were performed under local anesthesia and sterile conditions. After confirming satisfactory anesthesia, the ablation needle was precisely positioned at the pre-planned location. Currently, the energy output power and duration of MWA have not been standardized. In clinical practice, two main ablation modes are commonly used: one is a low-power, long-duration mode, and the other is a high-power, short-duration mode. We preset the ablation parameters based on the size, location, lung quality, and proportion of solid components in the lesion. Additionally, the patient's tolerance and the size of the target area play crucial roles during the ablation process, and these factors are used to appropriately adjust the final ablation parameters. The needle's position was verified using CT guidance before ablation was performed according to the pre-set power and time parameters. All MWA procedures were performed under local anesthesia and sterile conditions. After confirming satisfactory anesthesia, the ablation needle was precisely positioned at the pre-planned location. The needle's position was verified using CT guidance before ablation was performed according to the pre-set power and time parameters. The criteria for technical success were defined as achieving an ablation zone that extended 5–10 mm beyond the lesion boundary and matched the preoperative plan.

During the entire MWA procedure, patients' ECG and vital signs were closely monitored. Symptoms such as breathing difficulties, pain, coughing, and hemoptysis were observed, and symptomatic treatments were provided as necessary. After safely returning to the ward, patients were monitored for an additional 24 h. A follow-up chest CT scan was performed 24–48 h postoperatively to reassess technical success and detect any complications.

### 2.3 Equipment and VATS procedure

VATS was executed using the IMAGE1 HD video system (Karl Storz, Inc., Germany) and the Harmonic ultrasonic surgical scalpel (Ethicon Endo-Surgery, LLC, Puerto Rico, USA).

All surgeries were performed by our experienced thoracic surgery team to ensure complete tumor resection with negative margins while maintaining adequate safety margins. VATS was conducted under general anesthesia with single-lung ventilation. Patients were positioned in a full lateral decubitus position with the mid-chest level slightly over-extended to widen the intercostal space. A single 5 cm incision is made at the fourth or fifth intercostal space along the anterior axillary line. Through this incision, the mediastinal pleura and interlobar fissures are dissected, and the corresponding blood vessels and bronchi are separated during lobectomy or segmentectomy, following the same procedural steps as open surgery. An endoscopic linear cutting stapler is used to transect lung tissue, blood vessels, and bronchi, and a specimen retrieval bag is inserted through the small incision for extraction. Mediastinal lymph node dissection or sampling is performed simultaneously, and the resected primary lesion and lymph nodes are routinely sent for frozen section examination to complete lobectomy or segmentectomy. During wedge resection, the location of the lung nodule is determined based on preoperative imaging. The lung tissue is gently retracted to expose the target lesion, and if necessary, the pleural layers and interlobar fissures are separated. An endoscopic linear cutting stapler is then used to completely excise the lesion along with the surrounding lung tissue to ensure an adequate margin, while taking care to avoid damaging the lung vasculature and bronchi. During the procedure, the resected primary lesion is routinely sent for frozen section examination. Saline was instilled into the pleural cavity to expand the lung tissue, enabling assessment of the reliability of bronchial stump stapling, and checking for active bleeding or air leaks. At the end of the surgery, a 20F chest tube was inserted at the thoracic apex and connected to a negative pressure drainage system, and an 8F soft catheter was placed in the lateral chest wall for fluid drainage. The 20F chest tube was removed if no air leaks were detected and lung expansion was satisfactory 48 h postoperatively. The 8F chest tube was retained until discharge and was removed only when the drainage volume was less than 150 ml/day (16).

Patients' vital signs were closely monitored during the surgery. A chest x-ray or CT scan was performed within 24–48 h postoperatively to assess for complications.

### 2.4 Follow-up and outcome assessment

Chest CT scans were performed at 1, 3, and 6 months after MWA treatment, using the lesion at the first-month post-MWA as the baseline for comparison. Key assessments included whether the local lesion was completely ablated, recurrence of the local lesion, occurrence of new lesions in the lung, and incidence of complications. Subsequent CT scans were conducted every 6 months, transitioning to annual scans after 2 years (17). For patients who underwent VATS, follow-up intervals and outcomes were evaluated using the Response Evaluation Criteria in Solid Tumors (RECIST) (18).

The primary endpoint of this study was PFS, and the secondary endpoint was OS. PFS was defined as the time from the initiation of MWA or VATS treatment to the occurrence of any form of tumor progression or death. OS was defined as the time from the initiation of MWA or VATS treatment to death from any cause.

### 2.5 Assessment of complications

The study included common perioperative complications in thoracic surgery, assessing and grading their severity using the Clavien-Dindo classification system (19) This system categorizes postoperative complications into five grades: Grade I complications include the need for analgesics, antipyretics, antiemetics, electrolyte supplementation, and physical therapy, without requiring surgical, endoscopic, or radiologic interventions, but include wound infections requiring open drainage; Grade II complications require blood transfusion, total parenteral nutrition, or medications beyond those used in Grade I; Grade IIIa complications require surgical, endoscopic, or radiologic interventions without general anesthesia; while Grade IIIb requires such interventions under general anesthesia; Grade IVa involves life-threatening single organ dysfunction; Grade IVb involves life-threatening multi-organ dysfunction; Grade V denotes patient death. According to the Clavien-Dindo classification, Grade I complications generally do not require specific treatment. Thus, complications were further stratified into two categories: none or Grade I, and ≥Grade II.

### 2.6 Pulmonary function assessments

The study included several pulmonary function assessment indicators, including FVC, FEV1, FEV1%, MVV, and DLCO%. In the MWA group, pulmonary function data were recorded before the first lung resection surgery, pre-ablation, and post-ablation. In the VATS group, pulmonary function data were recorded before the first surgery and before the second.

### 2.7 Statistical analysis

Data analysis was performed using SPSS software version 26.0, while GraphPad Prism software (version 9) and the R software program were used to create charts. PSM was applied to balance potential confounding factors between the MWA and VATS groups. Categorical data were expressed as frequencies and percentages, with intergroup comparisons conducted using the *χ*2 test or Fisher's exact test. Continuous data were described using the mean ± standard deviation (x̅ ± s) or median (interquartile range) [M(Q1, Q3)] and compared between groups using the *T*-test or non-parametric tests. Kaplan–Meier methods were used to estimate PFS and OS, and survival outcomes were compared using the log-rank test. In univariate and multivariate survival analyses, Cox proportional hazards model were used to calculate hazard ratios (HR) and their 95% confidence intervals (CI). All statistical tests were two-sided, with a significance level set at *p* < 0.05.

## 3 Results

### 3.1 Patient characteristics

Between January 2021 and April 2024, 618 and 8,634 lung cancer patients underwent MWA treatment and lung resection surgery, respectively, at Peking University Cancer Hospital Yunnan Hospital. Based on strict inclusion and exclusion criteria, 220 and 335 patients were finally selected for the MWA and VATS groups, respectively. To balance the baseline characteristics of the two groups, 1:1 PSM was performed based on six variables significantly affecting prognosis: sex, age, TNM stage of the first surgery, FVC before the second treatment, FEV1 before the second treatment and SPLC lesion size (Table 1). The standardized mean difference (SMD) after matching is less than 0.2 (Figure 2). After matching, 202 patients were included in each group, achieving good comparability of clinical characteristics (Table 2).

**Table 1:** Characteristics of MWA and VATS groups before and after PSM.

Characteristics	Pre-matching				After matching			
	MWA group	VATS group	*P*	SMD	MWA group	VATS group	*P*	SMD
	(*n* = 220)	(*n* = 335)			(*n* = 202)	(*n* = 202)		
Age	56.49 ± 9.35	54.12 ± 8.52	0.002*	−0.278	55.67 ± 9.08	55.08 ± 8.32	0.5	−0.044
Gender			0.05	0.167			0.25	0.117
Male	77 (35%)	145 (43.28%)			75 (37.13%)	77 (38.12%)		
Female	143 (65%)	190 (56.72%)			127 (62.87%)	125 (61.88%)		
TNM stage of FPLC			0.248	0.124			0.81	0.044
IA	177 (80.45%)	259 (77.31%)			160 (79.21%)	171 (84.64%)		
IB	28 (12.73%)	32 (9.55%)			28 (13.86%)	14 (6.93%)		
IIA	3 (1.36%)	9 (2.69%)			3 (1.49%)	5 (2.48%)		
IIB	5 (2.27%)	14 (4.18%)			5 (2.48%)	6 (2.97%)		
IIIA	6 (2.72%)	19 (5.67%)			5 (2.48%)	5 (2.48%)		
IIIB	1 (0.45%)	2 (0.60%)			1 (0.50%)	1 (0.50%)		
Tumor size of SPLC (cm)	1.05 ± 0.52	1.44 ± 0.75	0.009*	0.211	1.14 ± 0.52	1.22 ± 0.45	0.18	0.128
FVC before the second treatment	2.48 ± 0.65	2.7 ± 0.68	<0.001*	0.323	2.53 ± 0.62	2.62 ± 0.64	0.139	0.149
FEV1 before the second treatment	2.1 ± 0.56	2.29 ± 0.59	<0.001*	0.332	2.18 ± 0.56	2.14 ± 0.48	0.122	0.153

Data are presented as number (%).

MWA, microwave ablation; VATS, video-assisted thoracoscopic surgery; PSM, propensity score matching; TNM, tumor node metastasis; FPLC, first primary lung cancer; SPLC, second primary lung cancer; FVC, forced vital capacity; FEV1, forced expiratory volume in 1 s.

* *p* < 0.05.

**Table 2:** Baseline characteristics of PSM population.

Characteristics	MWA group	VATS group	*p*-value
Total	202	202	
Age	55.67 ± 9.08	55.08 ± 8.32	0.50
Gender			0.25
Male	75 (37.13)	77 (38.12)	
Female	127 (62.87)	125 (61.88)	
Body mass index	22.86 ± 3.32	23.30 ± 3.07	0.17
Tuberculosis			>0.99
Yes	4 (1.98)	3 (1.49)	
No	198 (98.02)	199 (98.51)	
Smoking history			0.82
Yes	50 (24.75)	48 (23.76)	
No	152 (75.25)	154 (76.24)	
Diabetes			0.43
Yes	16 (7.92)	12 (5.94)	
No	186 (92.08)	190 (94.06)	
COPD			0.81
Yes	10 (4.95)	9 (4.46)	
No	192 (95.05)	193 (93.54)	
TNM stage of FPLC			0.81
IA	160 (79.21)	171 (84.64)	
IB	28 (13.86)	14 (6.93)	
IIA	3 (1.49)	5 (2.48)	
IIB	5 (2.48)	6 (2.97)	
IIIA	5 (2.48)	5 (2.48)	
IIIB	1 (0.50)	1 (0.50)	
Tumor size of SPLC (cm)	1.14 ± 0.51	1.17 ± 0.45	0.18
CTR of SPLC			0.64
≤50%	47 (23.27)	51 (25.25)	
>50%	155 (76.73)	151 (74.75)	
Histology type of SPLC			0.37
MIA	59 (29.21)	60 (29.70)	
IAC	139 (68.81)	137 (67.82)	
SCC	2 (0.99)	5 (2.48)	
PC	2 (0.99)	0	
Pulmonary function			
FVC	2.53 ± 0.62	2.62 ± 0.64	0.139
FEV1	2.18 ± 0.56	2.14 ± 0.48	0.122

Data are presented as number (%).

MWA, microwave ablation; VATS, video-assisted thoracoscopic surgery; COPD, chronic obstructive pulmonary disease; FPLC, first primary lung cancer; SPLC, second primary lung cancer; CTR, consolidation tumor ratio; MIA, minimally invasive adenocarcinoma; IAC, invasive adenocarcinoma; SCC, squamous cell carcinoma; PC, pulmonary carcinoid; TNM, tumor node metastasis; FVC, forced vital capacity; FEV1, forced expiratory volume in 1 s.

The mean age of patients in both groups was approximately 55 years, with the proportion of females significantly higher than males (MWA group: 62.87%, VATS group: 61.88%). The postoperative stage following the first lung resection was predominantly in the early stage, with over 90% of patients classified as stage IA. The mean diameter of SPLC lesions was 1.14 ± 0.51 cm in the MWA group and 1.17 ± 0.45 cm in the VATS group. Adenocarcinoma was the most common pathological type following both treatments. In the VATS group, 103 patients (50.9%) underwent lobectomy, and 99 patients (49.1%) underwent sublobar resection, including 50 patients (24.8%) who underwent segmentectomy and 49 patients (24.3%) who underwent wedge resection.

### 3.2 Survival outcomes

**Figure 3:**
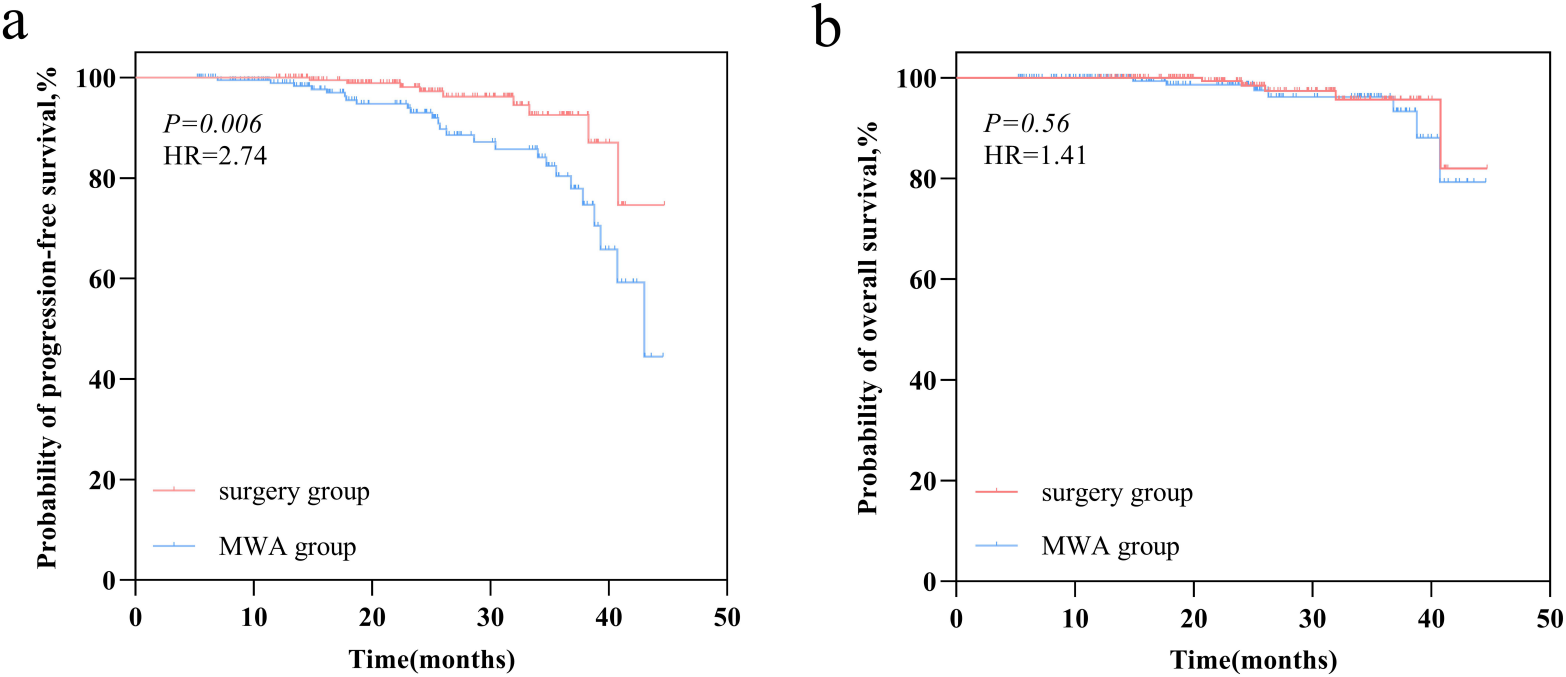
Survival outcomes comparison in the MWA group and VATS group. **(a)** Kaplan–Meier curve of progression-free survival (PFS). **(b)** Kaplan–Meier curve of overall survival (OS).

**Figure 4:**
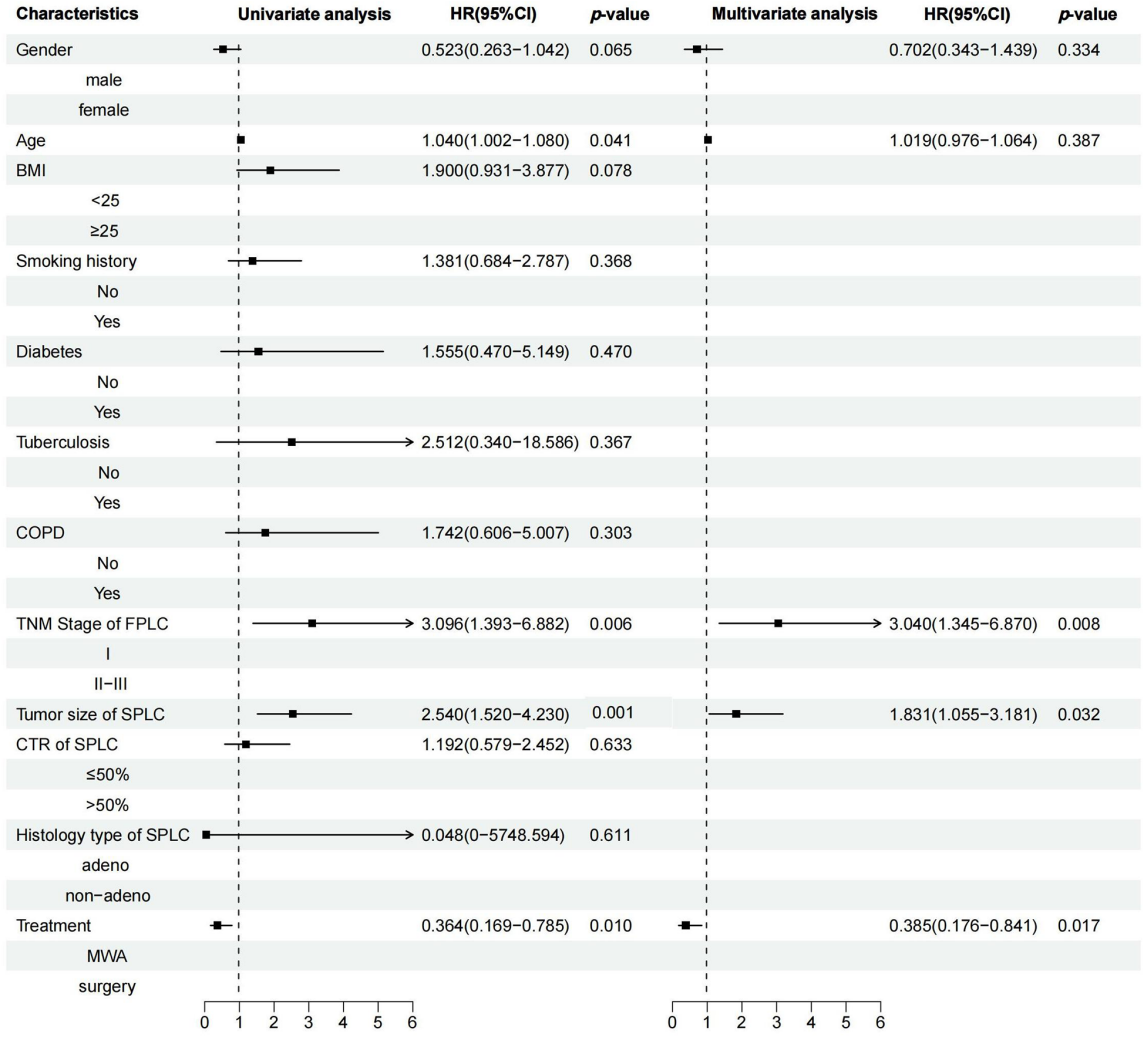
Univariate and multivariate Cox regression analyses of PFS in MPLC patients treated with MWA or surgery.

**Figure 5:**
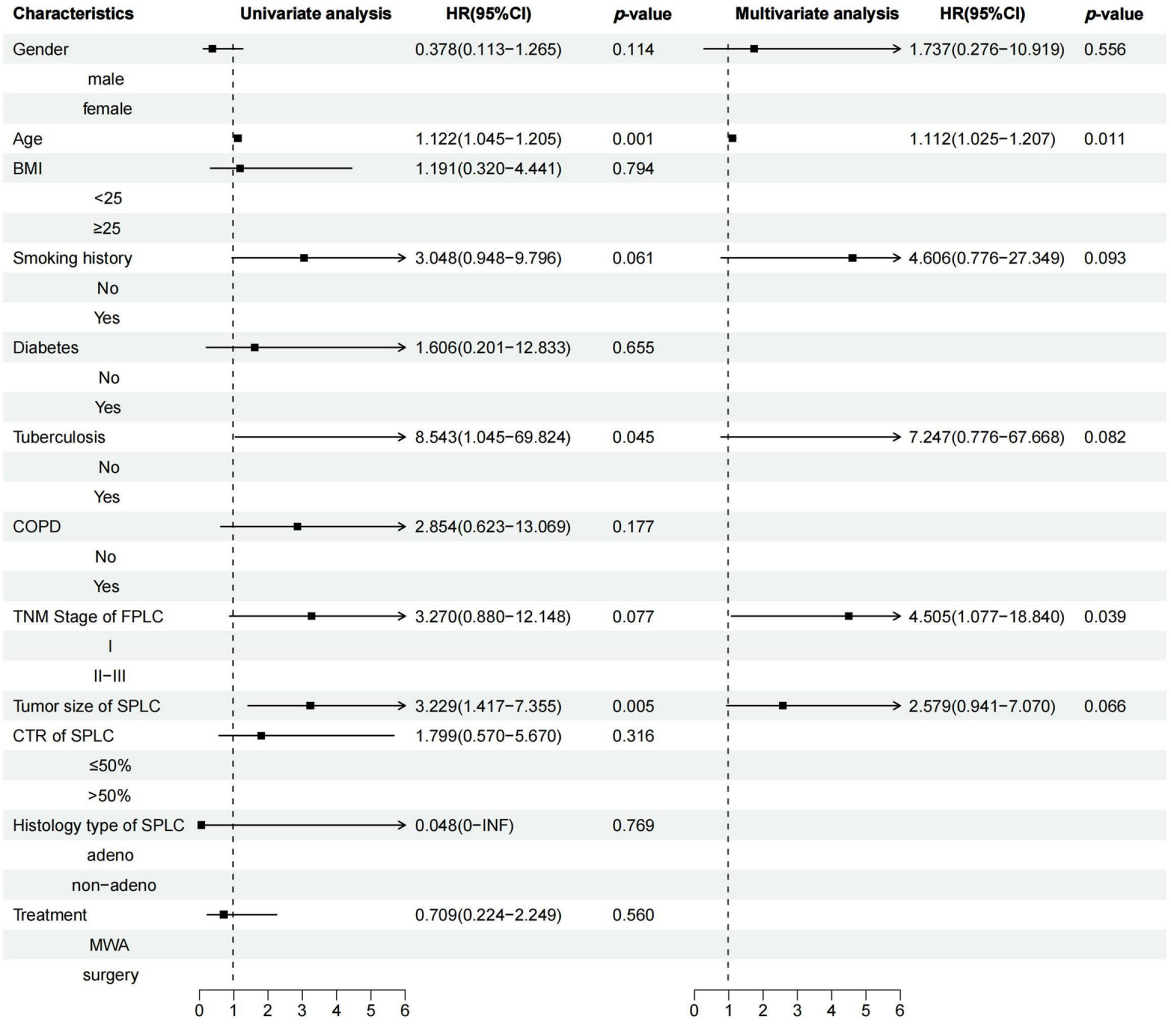
Univariate and multivariate Cox regression analyses of OS in MPLC patients treated with MWA or surgery.

Median follow-up was 24.47 months, ranging from 5.23–44.72 months. A statistically significant difference in PFS was observed between the MWA and VATS groups (HR = 2.74, 95% CI 1.40–5.36, *p* = 0.006) (Figure 3a), while the difference in OS was not statistically significant (HR = 1.41, 95% CI 0.45–4.36, *p* = 0.56) (Figure 3b). Univariate and multivariate survival analysis results indicated that MWA treatment, advanced TNM stage of FPLC, and larger tumor diameter of SPLC were independent risk factors for shorter PFS (Figure 4). The advanced TNM stage of FPLC and older age were identified as independent risk factors for shorter OS, but treatment modality was not an independent factor for OS (Figure 5). These findings suggest that while MWA has certain limitations in local tumor control, it remains an effective and reliable treatment option.

### 3.3 Complications

All patients successfully underwent MWA and VATS. In the MWA group, 48 patients (23.76%) experienced postoperative complications. The three most common complications were pneumothorax (37/56, 66.07%), pleural effusion (15/56, 26.79%), and pneumonia (3/56, 5.36%). In the VATS group, 45 patients (22.28%) experienced postoperative complications, primarily including pleural effusion (21/70, 30%), pneumothorax (14/70, 20%), and arrhythmia (15/70, 21.43%). No cases of multi-organ dysfunction or perioperative mortality occurred in either group. Comparative analysis of postoperative complications showed no statistically significant difference in overall complication rates (*P* = 0.133); however, the incidence of ≥Grade II complications was significantly different (*P* < 0.001) (Table 3), indicating that MWA-related complications were relatively less severe compared to VATS.

**Table 3:** The types and incidence of postoperative complications (cases).

Characteristics	Groups	Grade I	Grade II	Grade IIIa	Grade IIIb	Grade IVa	Grade I complications Incidence (%)	≥Grade II complications Incidence (%)
Pneumothorax	MWA group	28	9				13.9	4.5
	VATS group		11	3			0	6.9
Pleural effusion	MWA group	15					7.4	0
	VATS group		19	2			0	10.4
Chylothorax	MWA group						0	0
	VATS group				1		0	0.5
Active bleeding	MWA group						0	0
	VATS group		2		1		0	1.5
Pneumonia	MWA group		3				0	1.5
	VATS group		10				0	5
Bronchopleural fistula	MWA group						0	0
	VATS group				1		0	0.5
Thromboembolism	MWA group						0	0
	VATS group		2				0	1
Arrhythmia	MWA group						0	0
	VATS group		15				0	7.4
Heart failure	MWA group						0	0
	VATS group				1		0	0.5
Respiratory failure	MWA group						0	0
	VATS group				2		0	1
Cerebrovascular events	MWA group					1	0	0.5
	VATS group						0	0

Patients may experience ≥1 complication, corresponding to a ≥1 complication grade.

MWA, microwave ablation; VATS, video-assisted thoracoscopic surgery.

### 3.4 Pulmonary function

**Figure 6:**
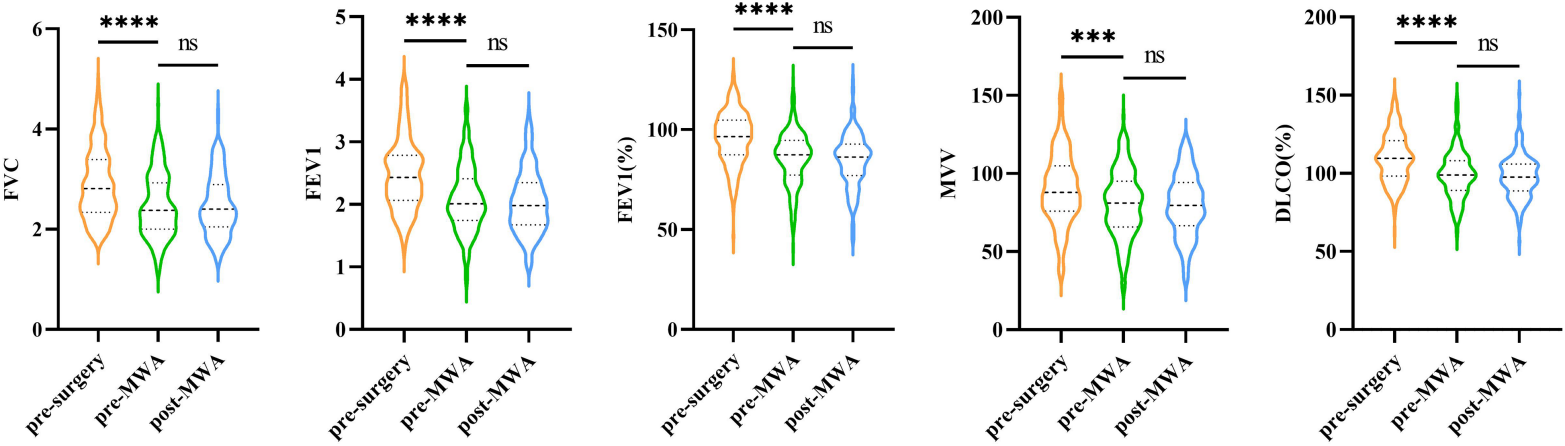
Changes in five pulmonary function parameters measured at three distinct time points (pre-surgery, pre-MWA, and post-MWA).

To effectively control inter-individual variability, this study compared pulmonary function changes in the MWA group at three-time points: before the first lung resection surgery, pre-MWA, and 1–3 months after MWA. The results showed that the five pulmonary function indicators: FVC, FEV1, FEV1%, MVV, and DLCO% had statistically significant overall differences across the three time points (*p* < 0.001) However, further group comparisons revealed no statistically significant difference between the pre-MWA and post-MWA measurements (Figure 6). These findings suggest that compared to surgery, pulmonary function remains relatively stable after MWA, demonstrating its protective effect on lung function.

## 4 Discussion

In the current study, we initially employed PSM to compare patients undergoing MWA and VATS. Subsequently, we conducted a systematic evaluation of the two groups, focusing on short-term efficacy, incidence of complications, and changes in pulmonary function. The results showed a statistically significant difference in PFS between the MWA and VATS groups (HR = 2.74, 95% CI 1.40–5.36, *p* = 0.006), with survival analysis indicating that MWA was an independent risk factor for shorter PFS. However, no statistically significant difference in OS was observed between the groups (HR = 1.41, 95% CI 0.45–4.36, *p* = 0.56), and MWA was not an independent factor for OS.

Regarding complications, pneumothorax was the most common complication in both groups. The proportion of patients with ≥Grade II complications was significantly lower in the MWA group, indicating better safety with MWA. there were no statistically significant differences in the five pulmonary function indicators before and after MWA treatment (*p* > 0.05), indicating a minimal impact on patients' pulmonary function.

With innovations in surgical techniques and approaches, VATS has become the mainstream treatment for early-stage NSCLC (20, 21). Meanwhile, the incidence of MPLC is increasing annually (22), but repeated surgeries may lead to high complication risks and restricted pulmonary function, posing clinical challenges (23). Fortunately, SBRT and ablation therapy have become important treatments for lung cancer (24). However, since MPLC typically involves multiple lesions with varying intervals of occurrence, combined with limitations in cumulative radiation doses and potential tumor resistance, these factors restrict the application of radiotherapy in MPLC (10, 25). In contrast, ablation therapy has demonstrated certain advantages in treating MPLC. Existing literature largely focuses on patients who opt for MWA due to poor cardiopulmonary function or other contraindications for surgery (26, 27). Currently, there is a lack of systematic comparative studies on MWA vs. surgical treatment for SPLC. The findings of studies would provide critical insights for optimizing treatment strategies for these patients.

Local ablation therapy has demonstrated good clinical efficacy and tolerability in clinical practice, making it a valuable treatment option for specific populations (28). MWA, in particular, has gained increasing research interest due to its high heating efficiency and low heat sink effect (14). A retrospective comparative study of MWA and lobectomy showed comparable 2-year OS and disease-free survival (DFS) between the two groups (27). Another study comparing MWA and sublobar resection for early subpleural NSCLC found no significant difference in 3-year OS but a slightly inferior recurrence-free survival (RFS) with MWA (23). Additionally, Han et al. (26). conducted a comparative analysis of 204 patients (101 of whom underwent MWA) with ground-glass opacity adenocarcinoma. The results showed comparable 3-year OS between the two groups, with no deaths observed and excellent local progression-free survival (LPFS). The results of this study showed no significant difference in OS between MWA and VATS, but VATS demonstrated superior PFS. The prognostic data in this study fell within the intermediate range of previous findings, being higher than that of patients with stage IA disease and severe cardiopulmonary dysfunction but lower than studies including only ground-glass nodules. The study also indicated that advanced TNM stage and larger lesion size are independent risk factors for MPLC progression, consistent with previous prognostic studies on lung cancer (29). The similarity in OS outcomes between VATS and MWA treatment may be related to the median follow-up time of 24.47 months in this study. Considering the relatively favorable prognosis of stage I lung cancer patients, this follow-up period may be too short to reveal potential differences between the two treatments in terms of long-term survival. The differences between the two treatment methods in terms of local tumor control may be attributed to the following factors. Firstly, VATS helps minimize the likelihood of residual disease by directly excising the tumor and confirming the surgical margins. In contrast, MWA is influenced by the heat sink effect and the tumor's anatomical location, which makes it difficult to precisely control the extent of thermal damage. This can potentially lead to incomplete ablation in certain areas, forming “sub-ablation zones” and increasing the risk of local recurrence. Secondly, compared to ablation, one significant advantage of surgery is the ability to perform systematic lymph node dissection or sampling, which not only helps identify and treat potential lymph node metastasis but also makes tumor staging more precise. In contrast, the staging in the MWA group primarily relies on imaging assessments (such as CT or PET-CT), with bronchoscopic biopsy or mediastinal/hilar lymph node sampling conducted in only a few suspicious cases. Therefore, some patients assessed as stage I by preoperative imaging and treated with ablation might actually be at stage II or higher, thus affecting the prognosis in the MWA group (30). Lastly, compared to surgical resection, MWA has certain limitations in assessing postoperative disease recurrence, particularly during early post-ablation imaging follow-up. The ablation target area is typically larger than the original tumor volume, as it includes both the tumor itself and the surrounding lung tissue affected by thermal damage. In a previous study, after 3 months of ablation, some patients may show increased fluorine 18 fluorodeoxyglucose (FDG) uptake on PET-CT scans, which in 15%–20% of cases was considered a response to treatment-induced inflammation rather than actual disease recurrence (31). Thus, this imaging change could lead to an overestimation of local recurrence rates and potentially affect the accurate assessment of MWA's therapeutic efficacy. In summary, for stage IA MPLC patients, surgical resection remains the first-line treatment. However, for patients with multiple, dispersed lesions or those who cannot tolerate or refuse lung resection, despite MWA having a higher risk of local recurrence compared to surgery, it remains an effective and viable alternative treatment option.

**SBRT has become the standard treatment for early-stage inoperable NSCLC.** However, its application in MPLC still faces several challenges. First, patients undergoing SBRT typically require an additional non-therapeutic invasive procedure to obtain a pathological diagnosis, whereas ablation therapy can acquire pathological results through simultaneous biopsy, which reduces patient discomfort and risk to some extent. Second, the lesions in MPLC present with complex characteristics, including synchronous and asynchronous onset, same-side same lung lobe, same-side different lung lobes, and bilateral lung lobes. These lesions are often located close together, exceeding the tolerance threshold for radiotherapy, which limits the clinical application of SBRT. Finally, radiation-induced lung toxicity (such as radiation pneumonitis and pulmonary fibrosis) remains a significant safety concern in thoracic radiotherapy and poses a major challenge for lung function preservation (32).

In terms of complications, the overall incidence of complications was similar between MWA and VATS (23.76% vs. 22.28%). Stratified analysis revealed that ≥Grade 2 complications were significantly more common in the VATS group, and the complication rate in the VATS group was consistent with post-MPLC surgery rates reported by other centers (15%–25.5%) (33, 34). It is worth noting that due to the lack of comparative data on Clavien-Dindo graded complications between MWA and surgical treatment in other studies, an effective comparison at specific grades could not be performed. However, Wang et al. (27). compared the overall complication rates of MWA and VATS, showing that complications in the VATS group were more severe than those in the MWA group, consistent with our findings. Pneumothorax, a primary complication of interest across studies, showed significant variation in incidence (15%–55%) (27, 28). In this study, the incidence of pneumothorax was 18.3%, which was well controlled, possibly due to strict pre-ablation planning and limitations on the number of puncture needles. Common postoperative complications, such as pneumothorax and arrhythmia, are easily detected through routine post-surgical examinations and clinical signs. The findings of this study suggest that most complications following MWA are self-limiting and do not require additional treatments such as medication or drainage. Therefore, based on previous research findings, patients who undergo MWA may experience shorter hospital stays and lower hospitalization costs (26, 27), while demonstrating a greater advantage in terms of safety.

Pulmonary function is an important indicator for evaluating the long-term quality of life in postoperative patients (35). Studies have shown that pulmonary function declines to some extent after VATS, and sublobar resection preserves pulmonary function better than lobectomy (34). In this study, all five pulmonary function indicators showed varying degrees of decline after VATS, consistent with previous research findings. Regarding MWA, Wu et al.'s study demonstrated that among 35 patients with pulmonary ground-glass nodules, pulmonary function experienced only a temporary decline after MWA and returned to baseline within six months (36). A retrospective study involving 133 MWA patients showed that pulmonary function could return to baseline levels within one month after ablation (37). The findings of this study showed no significant changes in pulmonary function indicators 1–3 months after MWA, aligning with previous literature. MPLC is a clinically complex and challenging subtype of lung cancer. However, there is currently no standardized surgical treatment guideline for MPLC or for residual and recurrent lesions following lung cancer surgery. Traditional surgical treatment often requires the resection of a significant portion of lung tissue, which inevitably increases perioperative complications and mortality rates. As the extent of surgical resection increases—from wedge resection to segmentectomy, and ultimately to lobectomy—progressive loss of lung function becomes inevitable. In particular, patients undergoing total pneumonectomy have a significantly higher likelihood of requiring home oxygen therapy postoperatively, which considerably impacts their quality of life. In contrast, a combined approach of surgery and MWA offers the advantage of preserving lung function while maintaining the potential for curative treatment. Therefore, MWA serves as a crucial therapeutic modality, offering enhanced lung parenchyma preservation and presenting a novel treatment option for MPLC patients who may otherwise suffer from substantial lung tissue loss due to multiple surgical interventions.

Our study had certain limitations that should be mentioned. First, the retrospective design of this study inevitably introduces potential biases, particularly in patient selection and data collection. Although baseline characteristics were balanced using propensity score matching (PSM), confounding effects from unobserved variables may still exist. To reduce such biases, future studies should prioritize prospective designs or randomized group assignments. Second, this study analyzed disease progression and survival times of patients after two treatments. However, as the data inclusion began in January 2021 and the follow-up time was relatively short, this may limit the comprehensive assessment of long-term outcomes. To further validate the long-term efficacy of microwave ablation (MWA), the research team plans to extend the follow-up period to provide more reliable and comprehensive efficacy data. Third, as a single-center study, the representativeness of the sample in this study may be limited, affecting the external validity of the results. To enhance the generalizability and reliability of the findings, future studies plan to conduct multi-center research to validate and extend the conclusions. Finally, The lack of post-second treatment pulmonary function data in the surgical group limits the comprehensiveness of the inter-group comparison, which may have a certain impact on the accuracy of the results.

In conclusion, for MPLC patients with stage IA SPLC, VATS demonstrates superior local tumor control compared to MWA and offers greater clinical efficacy. Additionally, MWA offers significant advantages over VATS in terms of complication severity and lung function preservation. These findings highlight the significant clinical value of MWA in the treatment of MPLC. Therefore, we recommend that clinicians consider MWA as a key adjunctive therapy in the treatment of MPLC when developing treatment plans, and optimize the strategy based on the individual needs of patients, particularly for those who require lung tissue preservation and seek to minimize surgical risk. Although this study is a single-center retrospective analysis, future multi-center studies and long-term follow-up will further validate and enhance the generalizability of these results.

## Data Availability

The raw data supporting the conclusions of this article will be made available by the authors, without undue reservation.
